# Antimicrobial Peptides against Multidrug-Resistant *Pseudomonas aeruginosa* Biofilm from Cystic Fibrosis Patients

**DOI:** 10.1021/acs.jmedchem.2c00270

**Published:** 2022-06-27

**Authors:** Daniel Ben Hur, Gal Kapach, Naiem Ahmad Wani, Edo Kiper, Moshe Ashkenazi, Gill Smollan, Natan Keller, Ori Efrati, Yechiel Shai

**Affiliations:** † Department of Biomolecular Sciences, Weizmann Institute of Science, Rehovot 76100, Israel; ‡ Pediatric Pulmonary Institute and National CF Center, Edmond and Lilly Safra Children’s Hospital, Sheba Medical Center, Tel Hashomer, Ramat Gan 52621, Israel; ∥ Sackler Faculty of Medicine, Tel-Aviv University, Tel-Aviv 69978, Israel; ¶ The Department of Health Management, Ariel University, Ariel 40700, Israel; § Microbiology Laboratories, Edmond and Lili Safra Children’s Hospital, Sheba Medical Center, Tel-Hashomer, Ariel University, Ramat Gan 52621, Israel

## Abstract

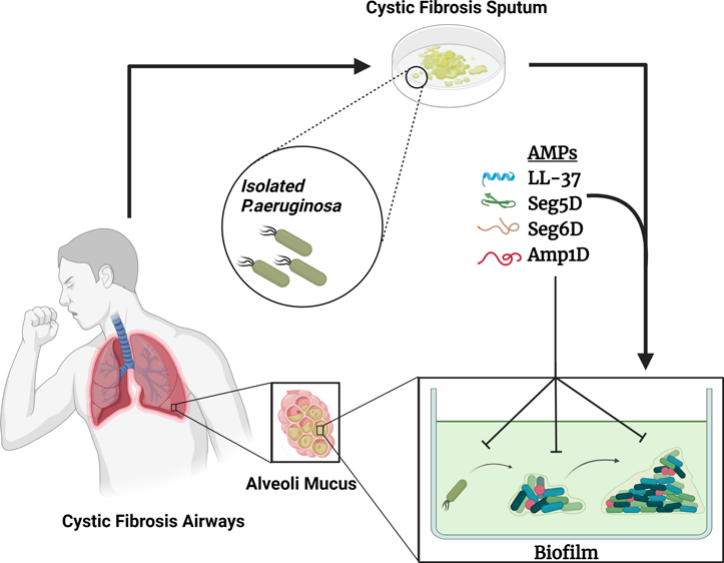

Lung infection is the leading cause of morbidity and mortality in cystic fibrosis (CF) patients and is mainly dominated by *Pseudomonas aeruginosa*. Treatment of CF-associated lung infections is problematic because the drugs are vulnerable to multidrug-resistant pathogens, many of which are major biofilm producers like *P. aeruginosa*. Antimicrobial peptides (AMPs) are essential components in all life forms and exhibit antimicrobial activity. Here we investigated a series of AMPs (d,l-K_6_L_9_), each composed of six lysines and nine leucines but differing in their sequence composed of l- and d-amino acids. The d,l-K_6_L_9_ peptides showed antimicrobial and antibiofilm activities against *P. aeruginosa* from CF patients. Furthermore, the data revealed that the d,l-K_6_L_9_ peptides are stable and resistant to degradation by CF sputum proteases and maintain their activity in a CF sputum environment. Additionally, the d,l-K_6_L_9_ peptides do not induce bacterial resistance. Overall, these findings should assist in the future development of alternative treatments against resistant bacterial biofilms.

## Introduction

Cystic fibrosis (CF) is an inherited disease that affects the respiratory system. The disease is triggered by a mutation in a gene that encodes the cystic fibrosis transmembrane conductance regulator (CFTR) protein, expressed primarily on epithelial cells.^1^ The CFTR regulates the transport of ions and the movement of water across the epithelial barrier.^2^ CFTR dysfunction causes a dehydrated thick mucus, promoting bacterial growth, leading to biofilm colonization, and affects components of innate immunity. The result is an exaggerated and ineffective airway inflammatory response, leading to airway tissue damage, eventually leading to respiratory failure.^3^ Chronic lung infections are the major cause of death among CF patients, and most of the infections involve colonization of the opportunistic multiresistant bacterium *Pseudomonas aeruginosa*.^4,5^*P. aeruginosa* developed resistance mechanisms toward antibiotics by decreasing antibiotic uptake, modifying enzymes, and developing a high rate of mutation and behavioral changes, which give the bacteria tools to fight against antibiotics and immune system components. Altogether, these factors allow the pathogen to survive and persist for years despite antibiotic treatments.^6−8^

*P. aeruginosa* specializes in forming sessile microcolonies that stick to a surface and each other, eventually forming a biofilm. These adherent cells are embedded within a self-produced matrix of extracellular polymeric substances (EPS), biofilm detachment cells, allowing biofilm formation on new surfaces. The EPS provide a barrier and protect the bacteria from harsh conditions such as host immune defense and antimicrobial agents.^9^

AMPs are part of the innate immune system in all living forms and serve as the first line of defense against pathogens.^10^ In humans, AMPs are stored in phagocytes in large quantities that can be released when invading microorganisms are encountered to stop microbial proliferation.^11^ AMPs share biophysical characteristics as they are short and composed of positively charged amino acids and hydrophobic amino acids but are not conserved in their sequences.^12,13^ Generally, AMPs are unstructured, potentially forming amphipathic α-helical or β-sheet structures in the membrane.^14,15^ AMPs disrupt the bacterial membranes without a specific high-affinity target and are assumed not to evolve resistance in pathogens, although some do.^15−19^ AMPs bind to the bacterial cell wall by electrostatic interactions with the anionic components. The hydrophobic interactions between the AMP and the bacterial acyl chains allow cell wall permeation due to the amphipathic structure that enables non-receptor-mediated attraction.^20,21^ Several AMPs were found to be active against planktonic bacteria and biofilm, e.g., LL-37, histatin, and nisin.^22−24^ However, these and many other AMPs were found to be toxic to eukaryotic cells at high concentrations; therefore, the discovery of new therapeutics is urgently needed.^24−26^*De novo*-designed AMPs that have high potency against bacterial cells and are not toxic to eukaryotic cells are promising candidates.^27−29^

Recently, we have designed a *de novo* peptide family named d,l-K_6_L_9_.^30^ Several modifications were made on the secondary structure by a different arrangement of the amino acids in the sequence and/or a partial replacement of l-to-d amino acids in peptides.^31−33^ These modifications improved their antimicrobial activity and resistance to protease degradation and reduced the level of hemolysis.^30,32,34^ In addition, the mechanism of killing of these peptides via membrane perturbation relies upon the inner bacterial membrane diffusion ability.^32^ Our work strives to understand the properties of AMPs that are required for efficient activity against CF clinical *P. aeruginosa* isolates, which sheds light on the vital properties of a new treatment for bacterial biofilm. Herein, the d,l-K_6_L_9_ peptides are active against PAO1 and CF isolates of *P. aeruginosa* at a planktonic stage and can inhibit and degrade biofilm in clinical and artificial CF sputum surroundings.

## Results

### Properties of the Antimicrobial and Antibiofilm AMPs

We investigated the antibiofilm and antimicrobial activities of a series of peptides containing nine leucines and six lysines each, with a net charge of +7 (Table 1). The parental peptide (Amp1L) has an α-helical structure and is toxic to mammalian cells.^32^ In contrast, Amp1D, Seg5D, and Seg6D, in which l-amino acids have been replaced with their d-enantiomers at positions 3, 6, 8, 9, and 13, resulted disruption in the secondary structure and hydrophobicity of the peptides (Table 1 and Figure S1).^30^ Similarly, the hydrophobic moment varies, which predicts an increase in the permeability of the AMP to the membrane^35^ (Table 1). Furthermore, in PE/PG phospholipid membranes and upon LPS interaction, it has been reported that these peptides have mixed α-helical, β-sheet, and random coil structures.^30,32^ Hydrophobic interactions stabilize the formation of a higher level of α-helix in Amp1D compared with those of the others.^30,32^ The biofilm inhibition and degradation mechanisms of the d,l-K_6_L_9_ peptides are surface adhesion, bacterial binding, and direct killing.^30^ Together with the d,l-K_6_L_9_ peptides, a well-known antimicrobial and antibiofilm peptide, human cathelicidin LL-37, was tested as a positive control (Table 1 and Figures S1 and S2). Amp1L, which consists of all of the l-amino acids of Amp1D, was used as a control for the protease stability of the d,l-K_6_L_9_ peptides in CF sputum (Table 1 and Figures S1 and S2).

**Table 1:** Peptide Designations and Properties

peptide designation	sequence^a^	length (no. of amino acids)	net charge	hydrophobicity (%AcN)^b^	hydrophobic moment^c^ (μH)
Amp1L	LKLLKKLLKKLLKLL-NH_2_	15	+7	67	0.835
Amp1D	LK**L**LK**K**L**LK**KLL**K**LL-NH_2_	15	+7	62.4	0.835
Seg5D	KK**K**LL**L**L**LL**LLL**K**KK-NH_2_	15	+7	60.2	0.191
Seg6D	LL**L**LL**K**K**KK**KKL**L**LL-NH_2_	15	+7	63.2	0.256
LL-37	LLGDFFRKSKEKIGKEFKRIVQRIKDFLRNLVPRTES-NH_2_	37	+8	71.6	0.521

a Amino acids shown in bold are d-enantiomers. All of the peptides are amidated on their C-termini.

b The peptides were eluted in a C18 reverse-phase analytical column. The duration of the elution was 40 min, using a linear gradient from 10% to 90% acetonitrile (AcN) in water, both containing 0.1% (v/v) TFA. The percentage of AcN was calculated according to the elution time.

c Hydrophobic moment (μH) of AMPs using HeliQuest (http://heliquest.ipmc.cnrs.fr).

### Selection of Clinically Isolated CF Patient *P. aeruginosa*

Thirty-one clinical *P. aeruginosa* multidrug-resistant (MDR) isolates from CF patients were collected by Tel-Ha’shomer hospital and verified as *P. aeruginosa* by MALDI-TOF mass spectrometry (Table S1). The isolates were tested for their sensitivity to different commercial antibiotics by BD-phoenix identification panels (Tables S1 and S2). The data included the sensitivity of each isolate to clinically used antibiotics and revealed the difference between the isolates in their sensitivity to the same antibiotic (Tables S1 and S2). We determined the antimicrobial activity of the AMPs only on CF *P. aeruginosa* clinical isolates that could grow under laboratory conditions. The isolates were taken to investigate the activity of the d,l-K_6_L_9_ peptides by a minimal inhibitory concentration (MIC) assay (Table S1).

### Activity of the AMPs against Clinical Isolates of *P. aeruginosa* from CF Patients

The AMPs listed in Table 1 were evaluated for their antimicrobial activity against planktonic bacteria using the MIC assay in a standard broth microdilution method. Human cathelicidin LL-37 and colistin were used as a positive control.^22^ The data revealed different MIC values for the different peptides (Table 2). LL-37 and Seg5D showed the highest diversity in their MICs among the tested isolates (Table 2). Although some isolates did not show a robust response (MIC > 25 μM), it is still considered a potent concentration. Seg6D showed antimicrobial activity (MIC ≤ 12.5 μM), and Amp1D was the most potent peptide against all of the tested isolates (MIC ≤ 3.12 μM) (Table 2).

**Table 2:** MIC_90_ Values (micromolar) of CF Patient Isolates of *P. aeruginosa*^a^

isolate no.	LL-37	Seg5D	Seg6D	Amp1D
24	12.5	25	12.5	3.12
25	3.12	6.25	6.25	1.56
29	1.56	6.25	1.56	1.56
40	12.5	25	12.5	1.56
46	1.56	3.12	1.56	0.78
52	1.56	12.5	6.25	1.56
53	6.25	6.25	6.25	1.56
59	6.25	25	6.25	1.56
71	12.5	25	12.5	3.12
72	6.25	25	12.5	3.12
82	1.56	3.12	1.56	1.56
94	6.25	25	12.5	3.12
95	1.56	3.12	1.56	0.78
99	3.12	25	12.5	1.56
172	1.56	25	6.25	1.56
238	25	25	6.25	3.12
251	1.56	6.25	3.12	1.56
629	12.5	25	12.5	3.12
995	12.5	6.25	6.25	3.12
PAO1	3.12	12.5	6.25	1.56

a The MIC (micromolar) was measured by a serial dilution method performed in a 96-well polystyrene plate. Plates were incubated for 24 h at 37 °C followed by absorbance (600 nm) measurements for bacterial growth.

### Inhibition of CF Biofilm Formation at Sub-inhibitory Peptide Concentrations

**Figure 1:**
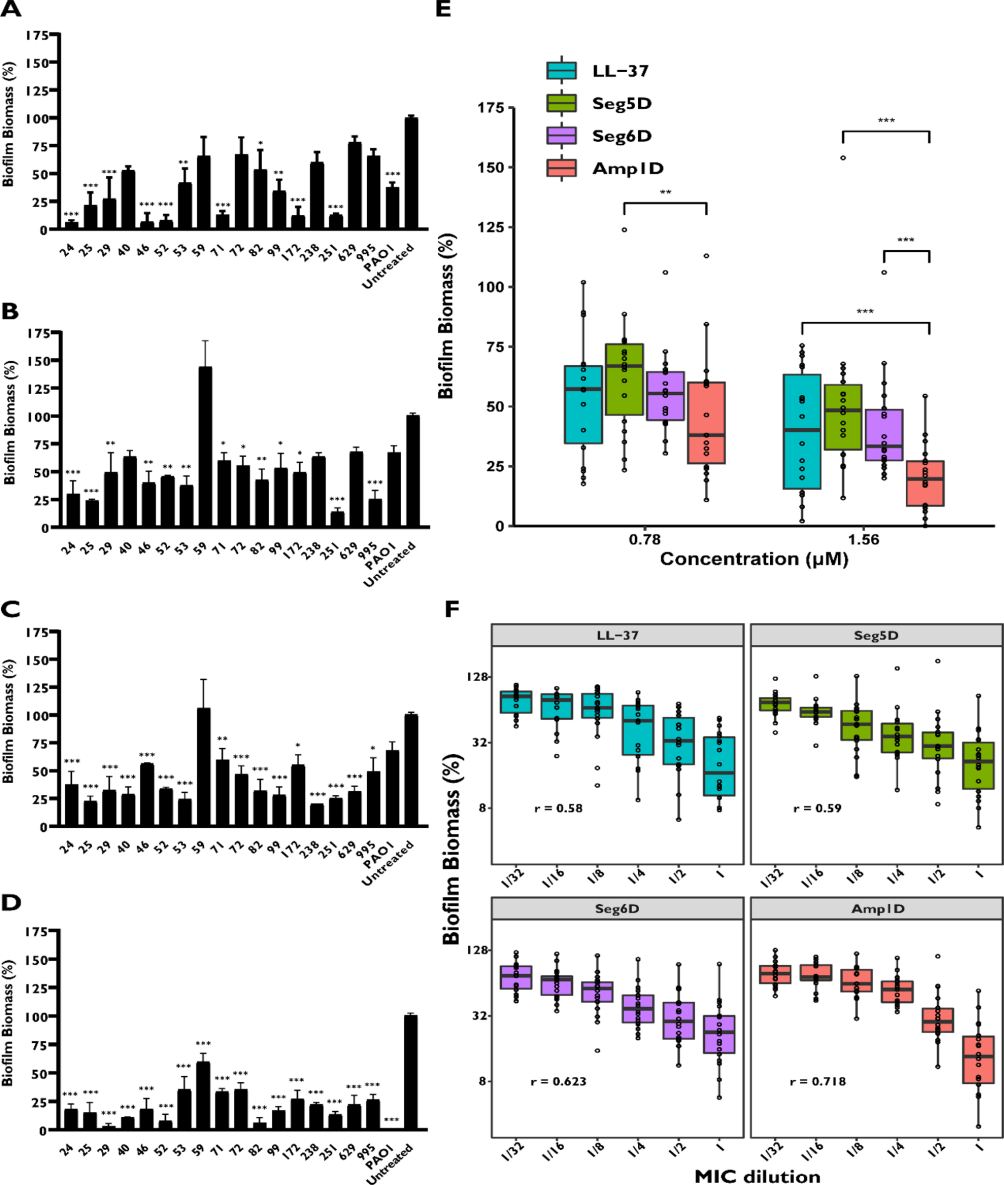
Inhibition of clinical CF isolates of *P. aeruginosa* biofilm formation in the presence of AMPs. *P. aeruginosa* bacteria were incubated for 24 h in the presence of AMPs. Biofilm after treatment was examined using 0.1% CV staining followed by absorbance measurements at 590 nm. The results are reported relative to untreated biofilm. Background measurements with no added bacteria were performed as blanks. (A–D) Biofilms were inhibited using 1.56 μM (A) LL-37, (B) Seg5D, (C) Seg6D, and (D) Amp1D. (E) Median of the biofilm biomass under 0.78 and 1.56 μM treatments. (F) Median of the biofilm biomass in MIC dilutions. Statistical significance determined by ANOVA. Correlation was tested by Pearson’s *R*.

Inhibition of biofilm formation was tested in a U-bottom microtitre 96-well plate without agitation to allow the bacteria to attach to the dish. Bacteria were allowed to form biofilm in the presence of the AMPs, and the biofilm biomass was quantified using the crystal violet (CV) staining method. The antibiofilm activity was tested at MIC and sub-MIC concentrations for 19 isolates (Figure 1 and Figure S3). Two isolates, 94 and 95, did not successfully form biofilm and therefore are not presented. The peptides at the MIC concentration inhibited the formation of all of the biofilms of the clinical isolates except for clinical isolate 59 (Figure S3). Our data revealed a high degree of diversity in antibiofilm activity against the various isolates. LL-37 and Amp1D inhibited biofilm formation of clinical isolate 59 by ∼30% and ∼50%, respectively, in terms of MIC concentration (Figure S3), while Seg5D and Seg6D lost their activity (Figure 1A–D). Amp1D reduced at least 40% of the biofilm biomass compared to that of the untreated form (Figure 1D). Moreover, Amp1D was found to inhibit biofilm in clinical isolates 24, 52, 53, and 71 and more at sub-inhibitory concentrations (Figure S3). For isolates 24, 25, 46, 53, 71, 72, 99, and 629, antibiofilm activity was observed at sub-inhibitory concentrations on all of the tested peptides (Figure S3). Interestingly, in isolates 24, 25, 46, 99, and 995, the inhibition of biofilm formation in all of the tested concentrations was maintained solely by Seg6D and Seg5D (Figure S3). From a micromolar concentration point of view, Amp1D was the most active AMP against most samples and was potent against all isolates at 1.56 μM (Figure 1A–D). A comparison of the inhibitory activity between the tested AMPs was made to analyze the most CF *P. aeruginosa* biofilm inhibitor AMP. Amp1D was found to be the most inhibitory AMP at 1.56 μM and maintained its activity in moderate mode once the concentration was reduced by half (Figure 1E). Moreover, AMPs were evaluated in a dose-dependent manner, revealing that Amp1D has the highest correlation (*r* = 0.718) between MIC dilution and biofilm inhibition (Figure 1F). However, LL-37 and Seg5D displayed similar correlations (*r* = 0.58 and *r* = 0.59, respectively), and Seg6D showed an *r* of 0.623 and validated that Seg5D and Seg6D preserve their activity at low concentrations (Figure 1F).

### d,l-K_6_L_9_ Peptides and LL-37 Degrade Established Biofilms of the Clinical Isolates

**Figure 2:**
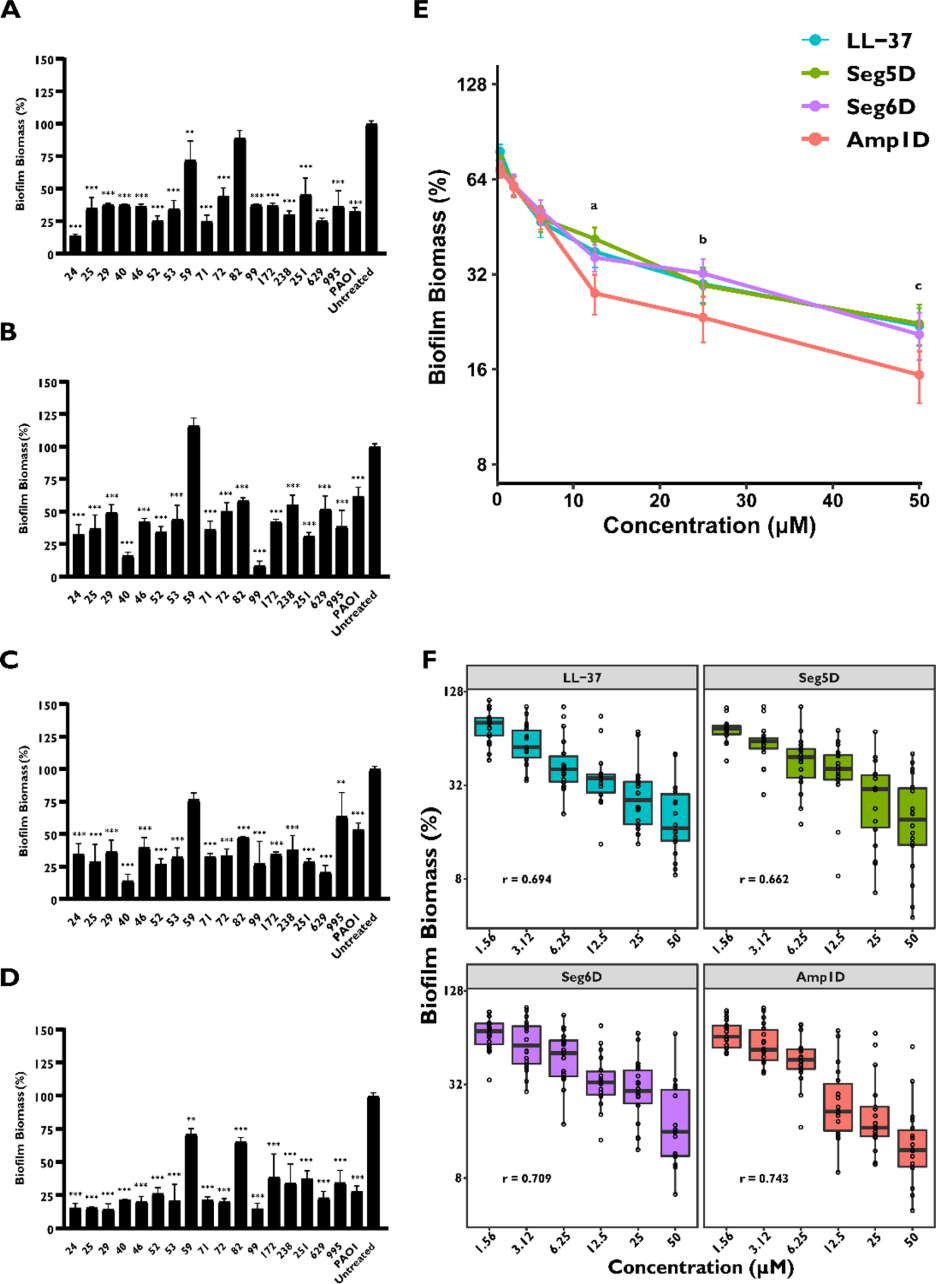
Clinically isolated CF patient *P. aeruginosa* biofilm degradation in the presence of AMPs. *P. aeruginosa* bacteria were allowed to grow for 24 h and treated for 1 h with peptides. Surface-associated biofilm after treatment, examined using 0.1% CV staining followed by absorbance measurements at 590 nm. The results are reported relative to untreated biofilm. Background measurements with no added bacteria were performed as blanks. Data represent a 12.5 μM treatment by AMPs (A) LL-37, (B) Seg5D, (C) Seg6D, and (D) Amp1D. (E) Comparison of biofilm degradation for treatments with 1.56–50 μM AMP. (F) Median of the biofilm degradation within the AMP treatment. The data are presented as means ± standard errors. Statistical significance from untreated biofilm was determined by ANOVA. The significant difference in the biofilm degradation comparison between AMPs was determined by the Dunnettx method. (a) Significant difference between LL-37 and Amp1D (*p* ≤ 0.01) and between Amp1D and Seg5D and Seg6D (*p* ≤ 0.001). (b) Significant difference between Amp1D and LL-37 (*p* ≤ 0.05) and between Amp1D and Seg6D (*p* ≤ 0.01). (c) Significant difference between Amp1D and LL-37 (*p* ≤ 0.001), between Amp1D and Seg5D (*p* ≤ 0.01), and between Amp1D and Seg6D (*p* ≤ 0.05). Correlation was tested by Pearson’s *R*.

We further investigated the activity of the AMPs to degrade established biofilms from CF *P. areganosa* clinical isolates. The biofilm was allowed to grow prior to the addition of peptides in serial dilutions. The biofilm biomass was evaluated using CV staining. The percentage of the biofilm biomass was different between isolates and peptides. Except for clinical isolate 59, all of the d,l-K_6_L_9_ peptides at 50 μM degrade the biofilm biomass by at least 60% (Figure S4). Seg5D, Seg6D, and LL-37 exhibited a lower biofilm degradation activity in isolate 59 of ∼40% (Figure S4). In contrast, Amp1D maintained its degradation activity at lower concentrations in isolate 59 (Figure S4). For clinical isolates 24, 40, 53, and others, degradation occurred at sub-MIC concentrations on all of the peptides (Figure S4 and Table 2). Seg5D showed the most efficient biofilm degradation activity at sub-MIC concentrations against all of the isolate biofilms except biofilm isolate 82 (Figure S4). Generally, all of the clinical isolate biofilms were degraded by at least two of three d,l-K_6_L_9_ peptides at their MIC values by 30% (apart from isolate 82). LL-37, Seg5D, and Seg6D degraded ∼50% of most established biofilm at 12.5 μM (Figure 2A–C). Amp1D degraded ∼75% of the established biofilm of most of the isolates at 12.5 μM (Figure 2D). In contrast to the inhibition results, LL-37 did not show an advantage in its degradation activity on isolated sample 59 compared to the other AMPs (Figure 2A). Amp1D was found to have the most potent and broad-spectrum biofilm degradation activity compared to those of other AMPs at high concentrations of 12.5–50–12 μM (Figure 2E). At concentrations of <12.5 μM, all AMPs demonstrated the same degradation level (Figure 2E).

Furthermore, AMP biofilm degradation was evaluated in a dose-dependent manner. We found that Amp1D has the highest correlation (*r* = 0.743) between the concentration and the biofilm degradation rate (Figure 2F). Seg6D displayed a correlation (*r* = 0.709) that was the same as that of LL-37 (*r* = 0.694), which are less dose-dependent on biofilm degradation. However, Seg5D was found to have the lowest dose-dependent manner among all des with an *r* = 0.662 correlation (Figure 2F). Overall, our findings suggested that d,l-K_6_L_9_ peptides possess biofilm inhibition and biofilm degradation activity against most of the clinical MDR isolates.

### Antibiofilm Activity of Amp1D against *P. aeruginosa* CF Isolate and PAO1 Biofilm in Artificial Sputum Media (ASM)

**Figure 3:**
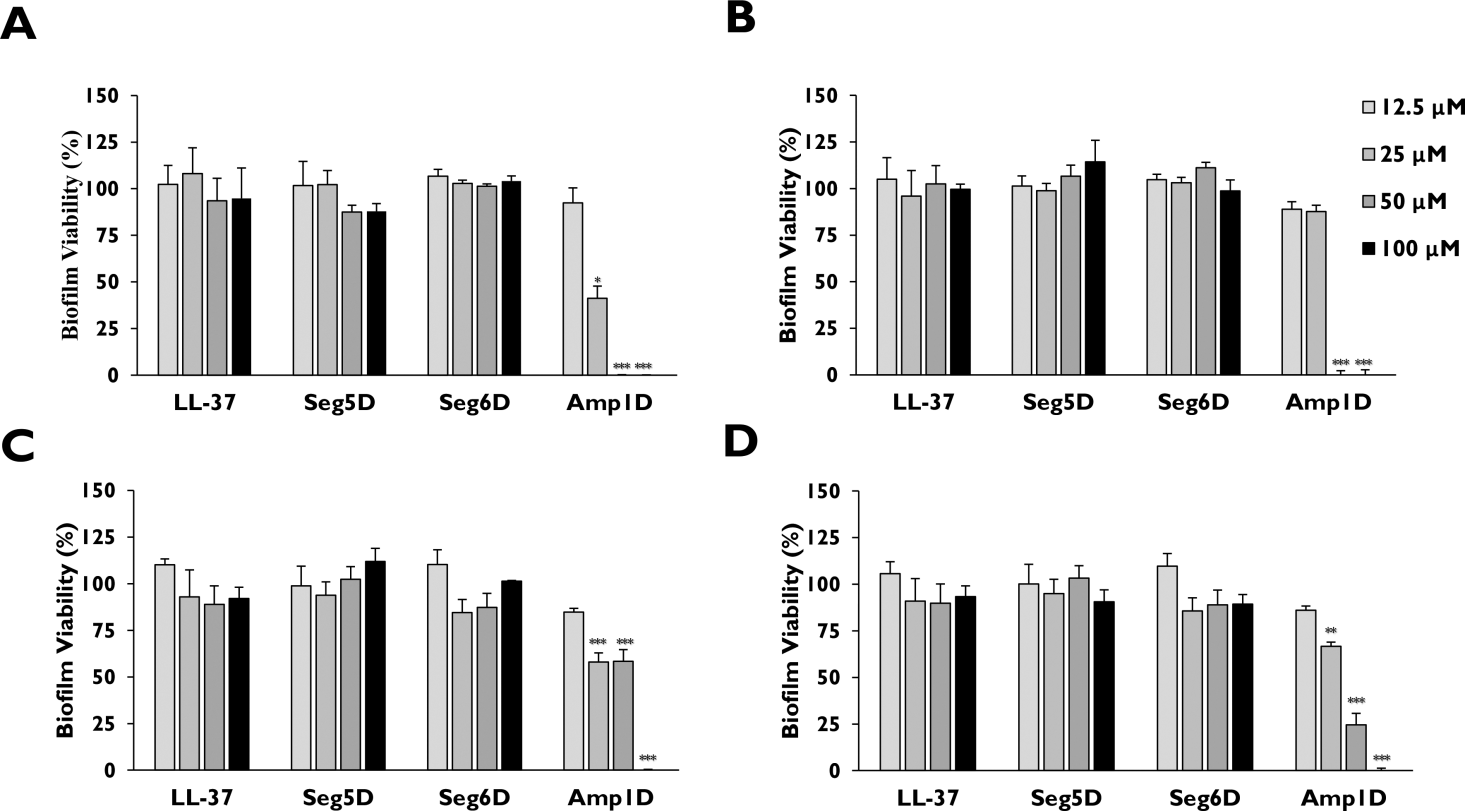
Effects of AMPs on *P. aeruginosa* CF isolate and PAO1 biofilm in ASM. *P. aeruginosa* CF isolates and PAO1 were grown in a 24-well plate with ASM for 3 days prior to exposure to different concentrations of AMPs for 24 h. Disruption of the biofilm was conducted by a 1 h incubation with cellulose. Viability was measured by a 2 h incubation with 0.02% resazurin. A plate reader with an excitation wavelength of 530 nm and an emission wavelength of 590 nm was used to measure fluorescence: (A) CF isolate 24, (B) CF isolate 46, (C) CF isolate 82, and (D) PAO1. The data are presented as means ± standard errors. The statistical significance from untreated biofilm was determined by ANOVA.

Artificial sputum medium (ASM) is a homogeneous and nonviscous culture medium containing the components of CF patient sputum composed of amino acids, mucin, free DNA, etc.^36−39^ ASM mimics the CF airway during *P. aeruginosa* infection, thus allowing the formation of self-aggregating biofilm structures and population variance. The ASM assay was tested against three CF clinical *P. aeruginosa* isolates and PAO1 (Figure 3). The three CF clinical *P. aeruginosa* isolates and PAO1 were allowed to grow for 72 h, and then the bacteria were treated with LL-37, Seg5D, and Seg6D for 24 h. After the treatment, we evaluated the biofilm viability using resazurin. Peptides LL-37, Seg5D, and Seg6D were less active in BM2 surroundings than in ASM surrounding at 12.5–100 μM (Figure 3A–D). Amp1D was found to be active against all CF isolates and PAO1 at 100 and 50 μM (Figure 3A–D). Isolates 24 and 82 and PAO1 were sensitive to Amp1D at 25 μM with 41.24%, 57.94%, and 66.7% biofilm viability, respectively (Figure 3A,C,D).

### Stability of the AMPs against Proteolysis in CF Sputum

**Figure 4:**
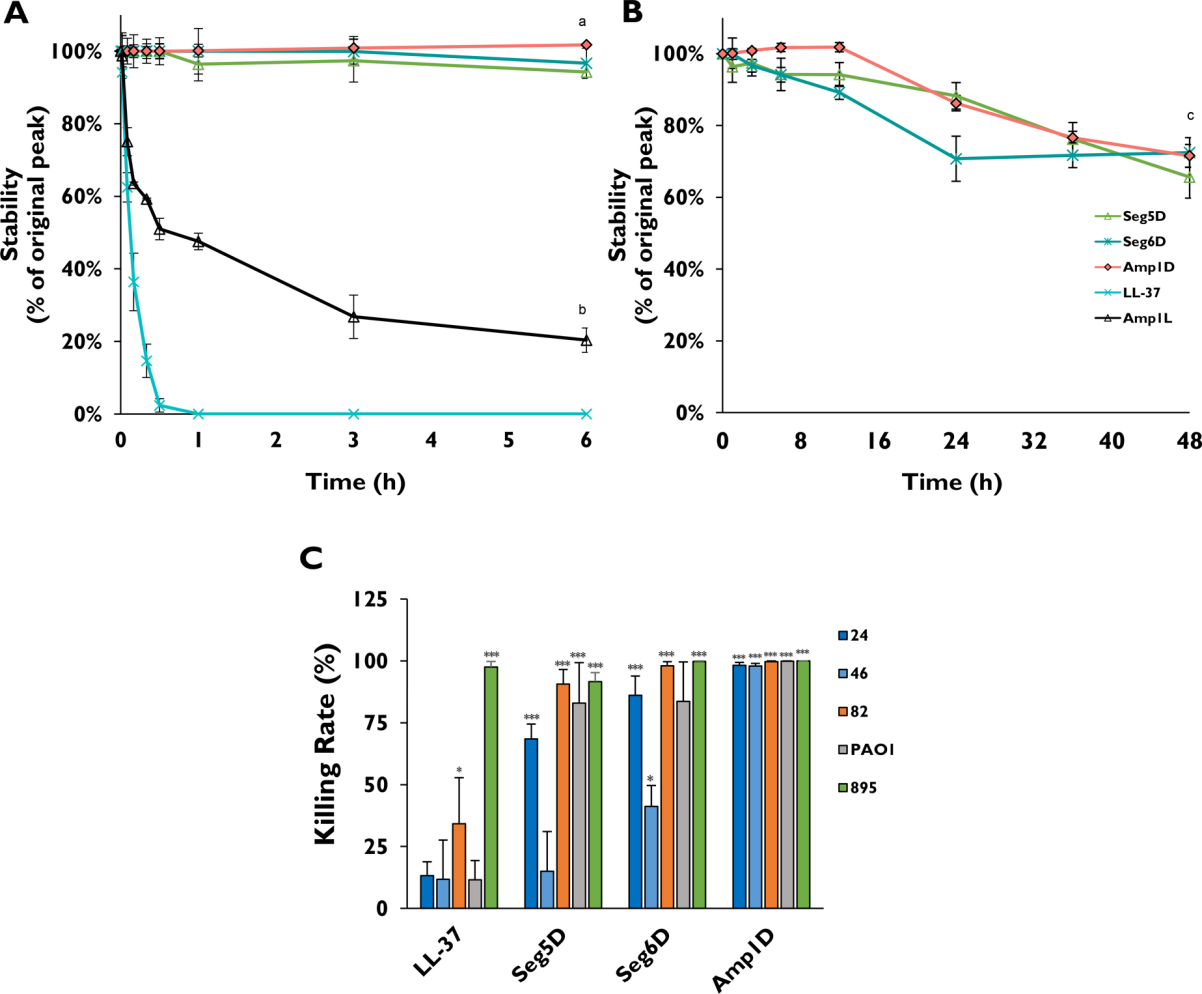
AMPs stability and activity on *P. aeruginosa* CF isolates and PAO1 in the presence of CF sputum. Sputum samples from CF patients were pooled and diluted in a 1:10 ratio with PBS (for stability and killing assay). K_6_L_9_ peptides and LL-37 were added to the supernatant to a final concentration of 100 μM, and the mixture was incubated at 37 °C for various time intervals. Residual peptide concentrations were determined by RP-HPLC as described in the Experimental Section. (A) d,l-K_6_L_9_ peptides, Amp1L, and LL-37 for 6 h. (B) d,l-K_6_L_9_ for 48 h. (C) Killing rate assays were performed on *P. aeruginosa* CF isolates and PAO1. Bactria were cultured in 10% CF sputum, exposed for 1 h to 10 μM AMPs, and plated on LB agar plates. The significant difference in the peptide stability assay was determined by a linear mixed model fit by REML, and *t* tests use Satterthwaite’s method. (a) Significant difference between the l-amino peptides and the d,l-K_6_L_9_ peptides (*p* ≤ 0.001). (b) Significant difference between Amp1L and the d,l-K_6_L_9_ and LL-37 peptides (*p* ≤ 0.001). (c) Significant difference between the d,l-K_6_L_9_ peptides (*p* ≤ 0.05). The killing rate statistical significance from the untreated biofilm was determined by ANOVA.

CF sputum in patients contains proteases, mucin, DNA, and ions. In addition, neutrophil elastase was found to be present at high concentrations and could degrade and deactivate antimicrobial peptides.^40^ We tested whether the d,l-peptides (Seg5D, Seg6D, and Amp1D) would demonstrate resistance to proteolytic degradation in CF sputum. The all-l-peptide (Amp1L) was used as a control as it shares the same amino acid composition as Amp1D but does not contain any d-amino acids (Table 1). The peptides were added to the diluted sputum to a final concentration of 100 μM, and the mixture was incubated at 37 °C for various time intervals. Using RP-HPLC, residual peptide concentrations were determined. First, we conducted the experiments for all of the peptides for 6 h. LL-37 was fully degraded after 30 min, and Amp1L was degraded by ∼70% after 3 h and eventually degraded by ∼80% after 6 h. In contrast, Amp1D, Seg5D, and Seg6D were protected entirely from degradation after 6 h (Figure 4A). Therefore, we extended the incubation time for these AMPs to 48 h. Seg5D and Amp1D showed similar degradation kinetics and were degraded by only ∼30% after 48 h (Figure 4B). Seg6D showed less moderate kinetics and was degraded by ∼30% after 24 h, which was maintained for the next 24 h (Figure 4B). In summary, the d,l-K_6_L_9_ peptides are significantly stable to proteolytic degradation and remain stable in the CF sputum environment.

### Bactericidal Activity of AMPs against *P. aeruginosa* CF Isolates and PAO1 in the Presence of CF Sputum

To test the activity of the peptides in an environment that mimics lungs of CF patients, sputum from CF patients was collected. *P. aeruginosa* CF isolates and PAO1 [each at 10^6^ colony-forming units (CFU)/mL] were added to diluted sputum together with 10 μM peptides. The mixture was then incubated at 37 °C for 1 h and plated on freshly made LB agar plates. The number of colonies was counted and compared to the number in the untreated plates. Isolate 895 was inoculated to 895 CF diluted sputum to eliminate the possibility of a certain element in the CF sputum that gives a specific isolate an advantage. LL-37 lost its activity in the presence of the mixed CF sputum with killing rates of 13.23%, 11.73%, 34.24%, and 11.48% toward isolates 24, 46, and 82 and PAO1, respectively. It was active only against control isolate 895 with a killing rate of 97.57% (Figure 4C). Seg5D was potent against isolates 24 and 82 and PAO1 with killing rates of 68.49%, 90.65%, and 82.93%, respectively. For isolate 46, the killing rate was only 15.01% (Figure 4C). The killing rates of Seg6D were 86.13%, 41.21%, 98.08%, and 83.64 toward isolates 24, 46, and 82 and PAO1, respectively (Figure 4C). Amp1D showed the highest killing rate of >97.91% against all of the CF isolates (Figure 4C).

### Visualizing *P. aeruginosa* PAO1 48 h Biofilm Distributed by Treatment with AMPs in the Presence of CF Sputum

**Figure 5:**
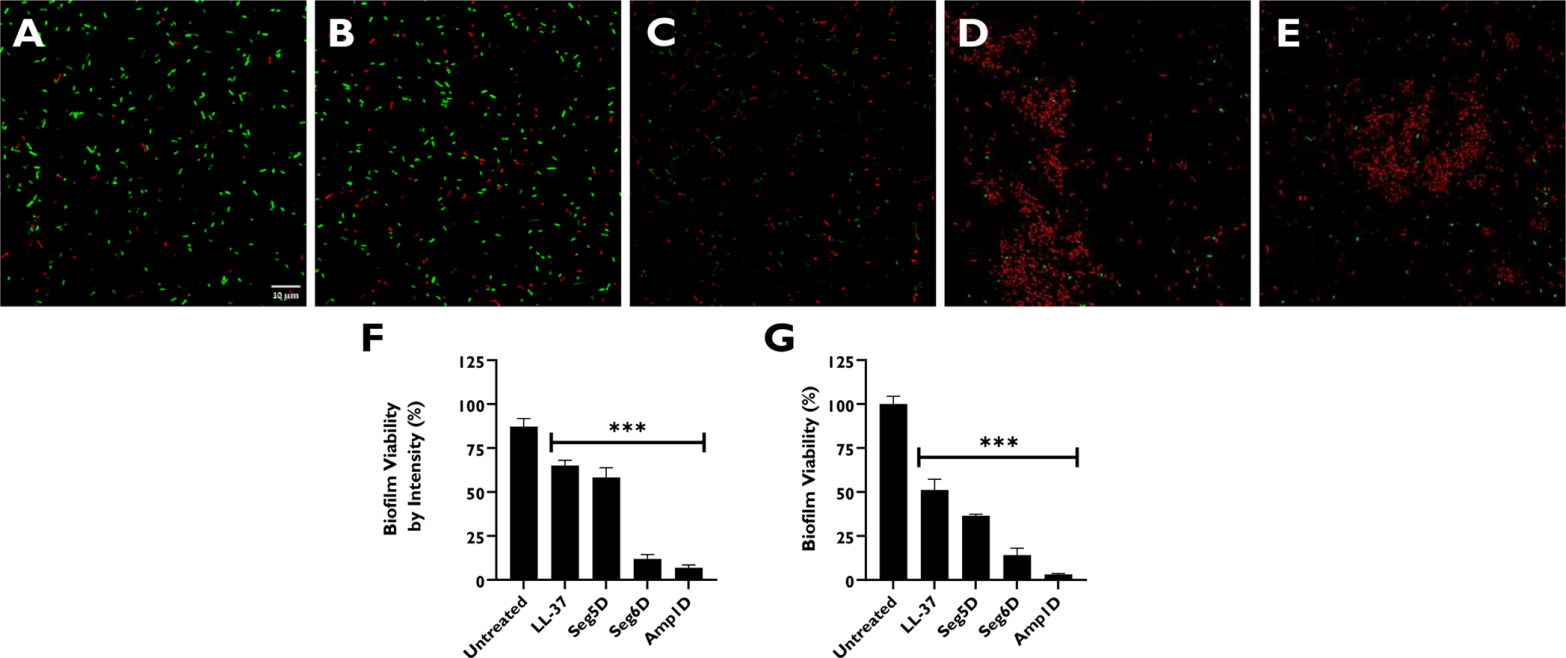
AMP antibiofilm activity in the presence of CF sputum. PAO1 was grown on a slide chamber with the sputum supernatant [10% (v/v)] for 48 h prior to exposure to a concentration of 10 μM for 1 h. Biofilms were stained with a live/dead cell kit for 1 h prior to fluorescence imaging: (A) untreated sample, (B) LL-37, (C) Seg5D, (D) Seg6D, and (E) Amp1D. Images were taken using an Olympus FV1000 confocal microscope [60× objective lens (oil), 10 μm scale bars]. (F) Biofilm viability according to fluorescence intensity. Data were analyzed using Olympus Fluoview (version 4.1) and ImageJ. (G) The viability of biofilms was checked using resazurin. The statistical significance from untreated biofilm was determined by ANOVA.

CF sputum substances can neutralize AMPs, and the accumulation of the sputum supernatant may increase the viscosity of biofilms and enhance their resistance. To investigate the degradation activity of AMPs against biofilms grown in the presence of the sputum supernatant collected from CF patients, we incubated the biofilms for 48 h with 10% CF sputum and replaced the media after every 12 h. Following a 48 h incubation, 10 μM AMPs were added for 1 h, and the biofilms were stained with a live/dead cell kit for 1 h.^41^ Fluorescence microscopy images showed that the untreated biofilms contained a high proportion of live bacteria (Syto-9) compared with dead ones (propidium iodide) (Figure 5A). Next, the biofilms were subjected to a viability test using resazurin. Amp1D showed the highest level of biofilm degradation, followed by Seg6D and then Seg5D (Figure 5C–E). LL-37 showed the weakest biofilm degradation capability in the presence of CF sputum (Figure 5B). Overall, Seg6D and Amp1D were active when added to 10% CF sputum. LL-37 lost its potency in the presence of CF sputum (Figure 5F). The effect of Seg5D was moderate in CF sputum compared to the effects of Seg6D and Amp1D. However, the used concentration was 10 μM, which is a sub-MIC concentration.

### d,l-K_6_L_9_ Peptides Inhibit and Degrade Established Biofilms of the Clinical Isolates in the Presence of CF Sputum

**Figure 6:**
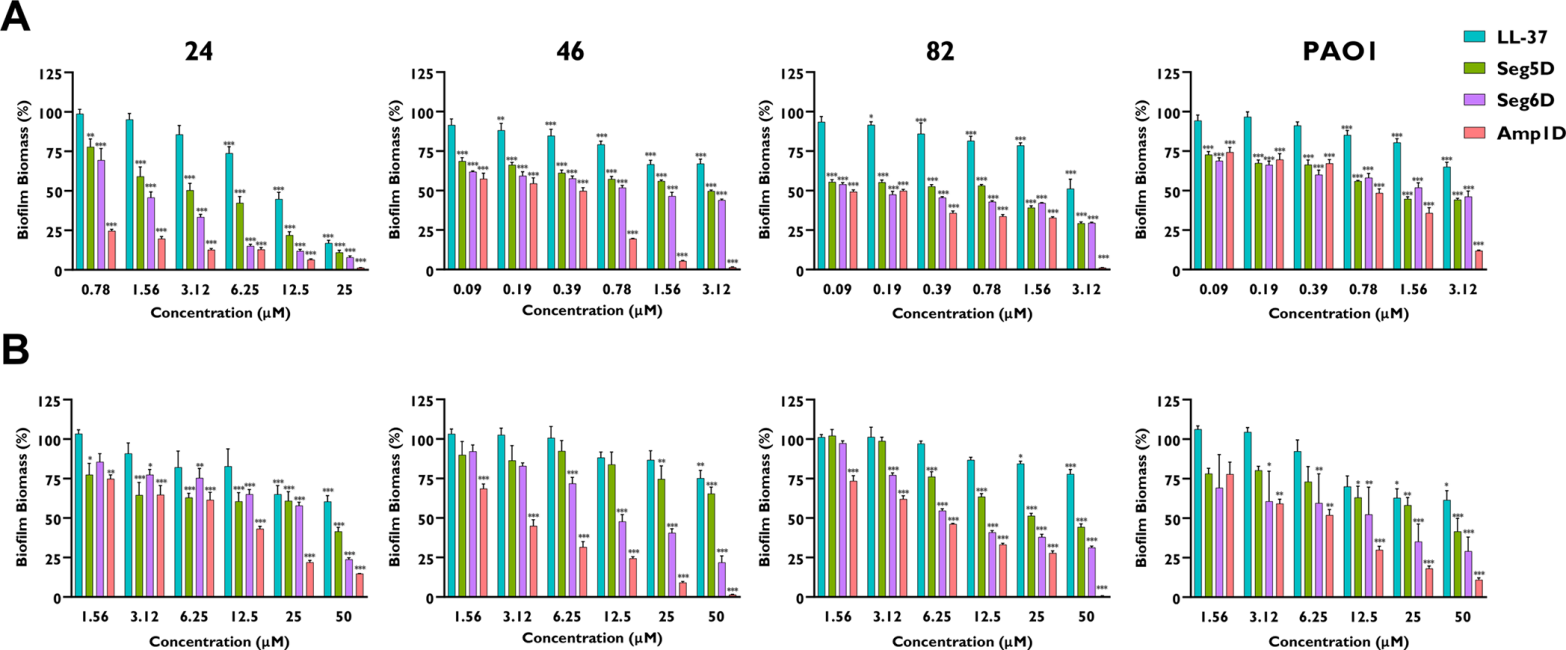
AMP inhibition and degradation activity against clinical CF isolate *P. aeruginosa* biofilms in the presence of CF sputum. A 1:10 BM2:CF sputum ratio was used for inhibition and degradation measurements. Surface-associated biofilm after treatment was examined using 0.1% CV staining followed by absorbance measurements at 590 nm. Results are reported relative to untreated biofilm. Background measurements with no added bacteria were performed as blanks. (A) Biofilm inhibition by AMPs using with the highest MIC for each isolate. (B) Biofilm degradation by AMPs in the treatment range of 50–1.56 μM. The statistical significance from untreated biofilm was determined by ANOVA.

Inhibition and degradation of the formation of CF clinical *P. aeruginosa* isolate and PAO1 biofilms in the presence of CF sputum were tested in an *ex vivo* model. The peptide biofilm inhibitory activity was evaluated starting with the highest MIC concentrations. To imitate the CF lung environment, the mixed sputum from CF patients was diluted and inoculated with selected CF clinical *P. aeruginosa* isolates and PAO1. All of the d,l-K_6_L_9_ peptides significantly inhibit biofilm formation (Figure 6A). The synthetic AMPs preserved their inhibitory activity in CF sputum while LL-37 lost its activity as the concentration decreased (Figure 6A). Amp1D was the most potent peptide among all of the peptides tested. However, as the concentration decreased, it showed biofilm inhibition like those of Seg5D and Seg6D (except isolate 24), as observed in the biofilm inhibition assay in BM2 (Figure 6A). Remarkably, d,l-K_6_L_9_ peptides maintain their biofilm inhibitory activity even at the lowest concentration. As observed in biofilm inhibition, the d,l-K_6_L_9_ peptides preserve degradation activity while LL-37 reduced it (Figure 6A). Amp1D was found to significantly degrade biofilm of all of the CF isolates and PAO1 at all of the tested concentrations, except PAO1 at 1.56 μM (Figure 6B). Thus, Amp1D significantly degraded biofilm from CF *P. aeruginosa* isolates at the MIC concentration compared to LL-37 (Figure 6B), while Seg5D and Seg6D have the same degradation rate against the CF isolates and PAO1 (Figure 6B).

### *P. aeruginosa* CF Isolates Do Not Evolve Constitutive Resistance to AMPs

**Figure 7:**
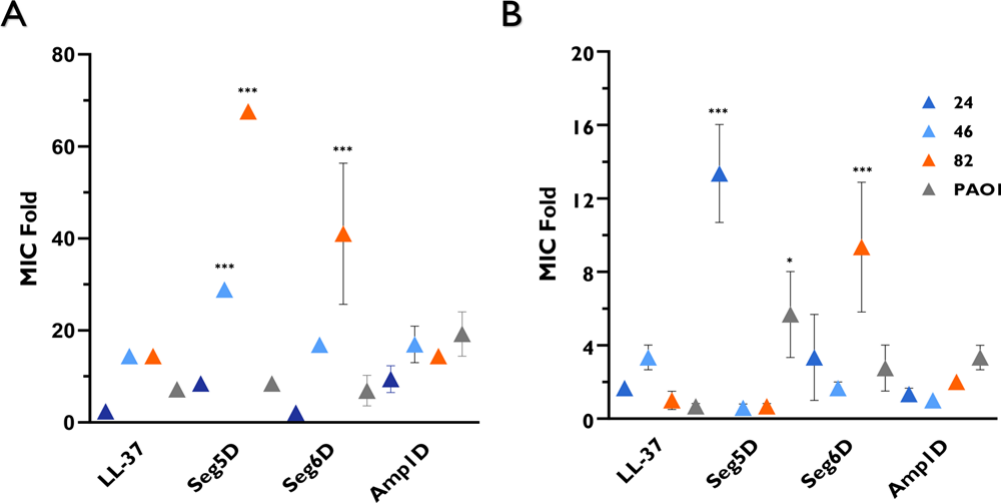
*P. aeruginosa* CF isolates and PAO1 do not evolve constitutive resistance to AMPs. *P. aeruginosa* CF isolates and PAO1 were serially propagated 45 times starting below the MICs of AMPs, which were increased. Growth was monitored by daily OD measurements. (A) MIC folds at the end of 45 parallel lineages. (B) MIC folds of constitutive resistance. The wells with the highest concentration were grown three times in fresh medium to determine their MIC after being washed with fresh medium. Statistical significance from DDW-treated *P. aeruginosa* determined by ANOVA.

Conventional antibiotic treatment frequently leads to the evolution of MDR bacteria. Resistance to AMPs is less common,^19^ but in some cases, it can be evolved by several mechanisms, including proteolytic degradation,^42^ surface modifications,^7^ and biofilm formation.^19,43^ We examined the ability of *P. aeruginosa* CF isolates to evolve resistance to d,l-K_6_L_9_ peptides and LL-37. The selected *P. aeruginosa* CF isolate and PAO1 bacteria from previous tests were exposed to four different AMPs (Table 1). Then, 45 parallel lineages with daily transfers were serially propagated in BM2 medium with increasing concentrations of the peptides. Cells that grew were transferred from the well into the fresh medium with increased concentrations of peptides (Figure 7A). The *P. aeruginosa* CF isolate resistance rate is represented by MIC folds, calculating the change in MIC. After prolonged exposure of 45 parallel lineages, the MIC values of most CF isolates and PAO1 increased but by <20-fold from the initial MIC except for isolate 46 to Seg5D and isolate 82 to Seg5D and Seg6D (Figure 7A). To determine if the resistance is constitutive, wells that reached the highest concentration were grown via three passages in fresh medium in the absence of peptides and were taken to assess their MICs (Figure 7B). All of the tested bacteria restored their initial MIC or close to it except isolate 24, with an MIC 12.02-fold that of Seg5D, and isolate 82, with an MIC 6.87-fold that of Seg6D. PAO1 generates hyposensitivity toward Seg5D with a 3.34-fold MIC. In general, it was demonstrated that after prolonged exposure to AMPs, the CF isolates and PAO1 induce temporary and low resistance but did not develop constitutive resistance.

## Discussion

To search for new potent CF sputum alternative antimicrobial agents, we investigated a family of d,l-K_6_L_9_ AMPs for their antimicrobial and antibiofilm activities against *P. aeruginosa* clinical isolates from CF patients. The CF *P. aeruginosa* isolates were found to be resistant to commercial antibiotics, which emphasizes the urgent need for new alternative drugs. All AMPs demonstrated antimicrobial activity against all CF isolates in the planktonic stage in BM2 surroundings. Although LL-37 is a promising candidate for dealing with bacterial biofilm infections in the clinic, it has low bioavailability and is toxic.^44^ We found that isolates highly resistant to commercial antibiotics were susceptible to the d,l-K_6_L_9_ peptides. For example, isolates 29 and 95, resistant to all antibiotics, are susceptible to our AMPs. Therefore, CF antibiotic-resistant isolates do not display cross-resistance to d,l-K_6_L_9_ peptides and LL-37. These data indicated that the mode of action comprises pathways different from those of the conventional antibiotics.^15^ Moreover, the peptides displayed an antibiofilm activity against all clinical isolates except isolate 59. When biofilms form, while there is stress, the AMPs might be a stressor that allows isolate 59 to grow biofilm biomass better than the untreated bacteria. Moreover, isolate 59 was the most pigmented isolate, which was caused by the production of pyocyanin (blue) and pyoverdine (green) virulence. Pyocyanin increases solution viscosity, and pyoverdine can create a cationic barrier by rejecting other cationic molecules.^45−47^ The antibacterial activity decreased in peptides Seg5D and Seg6D, which when tested at high concentrations may be due to the segregation of amino acids, which creates a charge cluster. In addition, the hydrophobic moment, which predicts an increase in the permeability of the AMPs to the membrane, increases in the following order: Seg5D < Seg6D < Amp1D. This explains the observed different activities (Table 1). However, the biofilm inhibition activity of Amp1D was found to fade at sub-inhibitory concentration, while Seg6D and Seg5D preserved their activity in the CF clinical *P. aeruginosa* isolates, indicating the importance of the ability of the AMPs to adhere to the bacteria to prevent biofilm formation at sub-inhibitory concentrations. When this is taken into consideration, the activities of these peptides are the same, once the peptides are penetrating the cell wall. These activity differences resulted from their diverse ability to diffuse through the bacterial cell wall.^32^ This phenomenon may occur in the biofilm, when the peptide concentration is low. Amp1D protrudes with its bactericidal activity and efficient biofilm inhibition and degradation. We assume that the biofilm degradation measurements were conducted following incubation with the AMPs for 1 h; considering the fact that the stability test showed that the peptides were stable for 48 h, it is possible that a longer incubation time would improve the effect of the d,l-K_6_L_9_ peptides. For further activity studies, we used ASM, which is a CF lung mimicking environment. In ASM, Amp1D degraded the biofilm while other AMPs lost their activity in the disruption of a 3 day biofilm. These findings highlight the importance of the positive charges spread along the AMPs to make the AMPs efficient in CF sputum. The lung environment of CF patients is rich in proteases that degrade native antimicrobial peptides. In contrast, the d,l-K_6_L_9_ peptides are stable in the sputum of CF patients. We found that the d,l-K_6_L_9_ AMPs are resistant to proteolytic degradation while all-l-amino acids peptides, Amp1L, and LL-37 are not. This partial replacement of l-to-d amino acids is sufficient to protect the peptide from degradation in the sputum. This property provides the peptides an advantage over l-peptides by extending their action time, which is reflected in better antibiofilm activity. We also observed that d,l-K_6_L_9_ peptides maintain their antibiofilm activity despite the presence of CF sputum, apart from Seg5D, whose MIC is higher than the used peptide concentration. In addition, we investigated the *P. aeruginosa* CF isolates to evolve resistance toward d,l-K_6_L_9_ peptides and LL-37. The data revealed that three selected clinical CF *P. aeruginosa* isolates and PAO1 generated moderate inducible resistance but did not induce constitutive resistance toward the AMPs. Isolate 82 induced hyposensitivity to Seg5D and Seg6D, which are the change cluster peptides, indicating the importance of the charge distribution in AMPs. It might be the key property to prevent the development of AMP resistance. d,l-K_6_L_9_ peptides maintain their antibiofilm activity in the presence of CF sputum due to their low level of proteolytic degradation toward proteases and low salt sensitivity. On the contrary, natural AMPs, such as LL-37, are unstable, sensitive to salt, and toxic. Because exogenic LL-37 loses its activity in CF sputum, treatment of patients with it may be detrimental, which can be reflected in other endogenous AMPs in natural surroundings.^40,48^ Combining all of the properties, our study suggests that synthetic peptides may be useful for treating CF-associated lung infections in contrast to LL-37. In summary, d,l-K_6_L_9_ peptides exhibited efficient antimicrobial activity against all CF isolates. Importantly, d,l-K_6_L_9_ peptides are more resistant to sputum proteases than l-peptides and do not induce constitutive AMP resistance. The possibility of inducing constitutive resistance was lower. Altogether, Amp1D as the most powerful AMP has antimicrobial and antibiofilm activity in CF sputum, is resistant to proteolytic degradation, and is quite unlikely to induce bacterial resistance. In addition to more than one AMP treatment, rotational medication/synergism in AMPs might be useful in preventing resistance and/or a combination therapy with antibiotics to enhance their therapeutic potential.

## Conclusions

We investigated a series of AMPs composed of six lysines and nine leucines with both l- and d-amino acids that differ in sequence. All of the peptides were tested against *P. aeruginosa* planktonic bacteria and biofilms isolated from CF patients. In addition of having strong antimicrobial and antibiofilm activity in the CF sputum environment, d,l-K_6_L_9_ peptides are stable in sputum and resistant to degradation by CF sputum proteases. Moreover, this peptide family is quite unlikely to induce resistance. The d,l-K_6_L_9_ peptides are short peptides that are easy to produce with a high yield of product, highlighting them as promising candidates for CF treatment.

## Experimental Section

### Chemicals and Materials

All of the reagents for the synthesis of the peptides were obtained from commercial sources and used without further purification. Fmoc amino acid coupling reagents [*N*,*N*′-diisopropylcarbodiimide (DIC) and ethyl cyanohydroxyiminoacetate (oxyma)] were purchased from Calibochem/Novabiochem AG (Laufelfinger, Switzerland). Trifluoroacetic acid (TFA), piperidine, and triisopropylsilane (TIS) were purchased from SigmaAldrich. Solvents for peptide synthesis, purification, and analysis, including *N*,*N*-dimethylformamide (DMF), dichloromethane (DCM), diethyl ether, and acetonitrile (ACN), were obtained from Bio-Lab Ltd. The rink amide resin (0.57 mmol/g) was purchased from Iris Biotech Gmbh, and the manufacturer’s reported loading was used to calculate the yields of the final product. Colistin and CV (C3886) were purchased from Sigma-Aldrich (Rehovot, Israel). Sterile 96-well U-bottom (BN36010096D) and flat bottom (BND003CSL) polystyrene plates were purchased from Bar-Naor (Ramat Gan, Israel). Basal Medium 2 (BM2) and Luria-Bertani (LB) medium were provided by the Weizmann Bacteriology Unit. Experiments were performed in at least three biological repeats with technical repeats.

### Peptide Synthesis and Cleavage

Peptides were synthesized by an automated peptide solid-phase synthesizer (CEM Liberty Blue peptide synthesizer) on rink amide 0.57 mmol/mg MBHA resin, using the Fmoc solid-phase strategy.^49^ To synthesize the peptides, Fmoc-Lys(Boc)-OH and Fmoc-Leu(Boc)-OH were used. The d-enantiomers of Fmoc-Lys(Boc)-OH and Fmoc-Leu(Boc)-OH in d,l-K_6_L_9_ peptides are involved at positions 3, 6, 8, 9, and 13. The resin-bound peptide was washed thoroughly with DMF and then DCM, dried, and cleaved. Cleavage was performed using 95% TFA, 2.5% water, and 2.5% TIS for 120 min at room temperature. The crude peptides were washed from the resin using TFA, precipitated using cold diethyl ether, and dried in air.

### Peptide Purification

The purification of the peptides was performed by reverse-phase high-performance liquid chromatography (RP-HPLC) on an Agilent Technologies 1200 Series instrument with a reversed-phase Vydac C18 column (Grace Discovery Sciences, 10 μm particle size, 250 mm × 10 mm) at a flow rate of 1.8 mL/min and monitored with an ultraviolet (UV) detector at 215 nm. Linear gradients of 20% to 90% acetonitrile in water containing 0.1% TFA were used for peptide purification for 40 min. Final products were obtained by freeze-drying the collected pure fractions.

### HPLC Analysis

The purity of the peptides was examined using RP-HPLC on an Agilent Technologies 1260 Infinity II spectrometer with a C18 reversed-phase column (Thermo Fisher Scientific, 250 mm × 4.6 mm, 5 μm particle size) at a flow rate of 0.6 mL/min using a gradient of 10% to 90% ACN in water containing 0.1% (v/v) TFA for 40 min with UV detection at 215 nm. The molecular masses of all of the peptides were determined by TOF-MS. The purity of all peptides examined for biological activity was >95%.

### Collection of Sputum from CF Patients and Isolation of *P. aeruginosa*

Sputum samples from 31 CF patients were collected and transferd to the bacteriology laboratory at Sheba hospitals center, Tel-Hashomer. All 31 samples were subcultured on blood agar plates (BAP) and transferred to LB plates. Then suspensions were stored at −80 °C in LB medium containing 17% glycerol. The samples were verified as *P. aeruginosa* by MALDI-TOF mass spectrometry using the Bruker Microflex LT, also called the MALDI Biotyper (MBT). The MALDI Biotyper (MBT) is an automatic device that uses the linear MALDI-TOF/MS technology to rapidly identify organisms on the basis of the mass of their proteins (mass spectrometry). Biomass from a colony is sampled and applied to 96 wells on a stainless steel target plate. A matrix of α-cyano 4-hydroxycinnamic acid (HCCA) dissolved in a solution containing a mixture of 2.5% trifluoroacetic acid, 50% acetonitrile, and 47.5% water was applied to the bacterial biomass on the target. A mixture of the matrix and sample co-crystallizes to form a solid deposit of the sample embedded in a matrix and loaded into the MALDI-TOF instrument. Additionally, this study used the *P. aeruginosa* PAO1 strain stored and grown under the same conditions as the clinical *P. aeruginosa* CF isolates.

### Identification of *P. aeruginosa* from CF Sputum

*P. aeruginosa* was identified by the BD Phoenix Automated Microbiology System (Becton Dickinson). The system is intended for the rapid identification (ID) and antimicrobial susceptibility testing (AST) of clinically significant bacteria. Tests used in the Phoenix ID panels comprise a 45-well ID side that includes tests for fermentation, oxidation, degradation, and hydrolysis of various substances and an 85-well side containing dried antimicrobial agents in coordination with the hospital formulary as QC and growth wells. The technique involves exposing bacteria to decreasing concentrations of antimicrobial agents. The Phoenix panels were inoculated according to the manufacturer’s instructions as follows. A standardized inoculum of 0.5 McFarland was prepared in Phoenix ID Broth, gently vortexed, and measured using the BD PhoenixSpec nephelometer to ensure the correct inoculum; then, 25 μL of the inoculum was added to the Phoenix AST broth after the addition of one drop of the Phoenix AST indicator solution. Once inoculated with the solutions from both tubes, the panels were placed in the Phoenix instrument and continuously incubated at 35 °C. The instrument reads the panels every 20 min for ≤16 h. The final ID and AST results are transferred via the EpiCenter to the LIS.

### Antibacterial Activity

The MIC of the peptides was tested as previously described with minor adjustments.^50,51^ Briefly, peptide activity was examined in sterile 96-well polystyrene plates (Bar-Naor BN36010096D). Overnight cultures of *P. aeruginosa* were washed and resuspended in a BM2 medium. Aliquots of 50 μL of suspended bacteria (1 × 10^6^ CFU/mL) were added to 50 μL of BM2 medium containing peptides in serial 2-fold dilutions (final concentration between 0.78 and 50 μM). Plates were incubated for 24 h at 37 °C while being agitated. Inhibition of growth was assessed by absorbance measurements at OD_600_ using a microplate auto reader. The MIC was defined as the concentration at which 90% inhibition of growth was observed after incubation for 24 h.

### Biofilm Inhibition

The biofilm formation assay was performed as described for the MIC assay with some alterations and used either BM2 or CF patient sputum diluted 10-fold with BM2. Plates containing peptides at serial dilutions and bacteria were incubated for 24 h at 37 °C without agitation to allow biofilm formation. Unattached bacteria were then washed from plates, and the plates stained with 0.1% CV, followed by absorbance measurements at OD_590_ using a microplate auto reader. Data are presented as a percentage of the biofilm biomass compared to biofilm formed by untreated bacteria ± the standard error.

### Biofilm Degradation

Degradation of biofilm was tested as described above for the MIC assay with some alterations. The medium was either BM2 medium or CF patient sputum diluted 10-fold with BM2. Plates containing 1 × 10^6^ CFU/mL were incubated at 37 °C without agitation to form biofilm. After being incubated for 24 h, all wells were rinsed to remove unattached bacteria and supplemented with serial dilutions of the d,l-K_6_L_9_ peptides and LL-37 (from 1.56 to 50 μM). After being exposed to the peptides for 1 h, wells were washed and examined using CV staining at 590 nm, as mentioned above. The results are presented as a percentage of the biofilm biomass compared to biofilm formed by untreated bacteria ± standard errors.

### Artificial Sputum Media Assays (ASMs)

Assays were performed in a 24-well plate using three *P. aeruginosa* CF isolated strains and PAO1 used as a reference strain. ASM is CF patient sputum mimic medium. The assay was conducted as reported previously.^36−39^ Briefly, 4 g of salmon sperm DNA and 5 g of mucin from porcine stomach were slowly dissolved overnight in 250 mL of sterile water. Then, the solution was combined with 0.25 g of each essential and nonessential l-amino acid in 100 mL of sterile water (except l-cysteine, which was dissolved in 25 mL of 0.5 M potassium hydroxide, and l-tyrosine, which was dissolved in 25 mL of sterile water), 5.9 mg of diethylenetriaminepentaacetic acid (DTPA), 5 g of NaCl, 2.2 g of KCl dissolved in 100 mL of sterile water, and 5 mL of egg yolk emulsion. The pH was adjusted to 6.9 with 1 M Tris (pH 8.5). The volume was increased to 1 L with sterile water, and then the mixture filtered using a vacuum pump and Millipore Steritop filter units with a pore diameter of 0.22 μm. Overnight cultures of *P. aeruginosa* were washed and resuspended in BM2 to an OD of ∼0.05 and then diluted 1:100 in fresh ASM. The total volume in each well was 1.8 mL. Plates were incubated for 3 days aerobically at 37 °C with agitation, allowing the *P. aeruginosa* biofilms to develop. After 3 days, 0.2 mL of AMPs was added at concentrations ranging from 12.5 to 100 μM and incubated for 24 h while being agitated. The biofilm was disrupted by cellulase [100 μL of a 100 mg/mL solution (pH 4.6, diluted in 9.6 g/L citrate)] and then incubated for 1 h at 37 °C while being agitated. For each well, resazurin was added [100 μL of a 0.02% (v/v) solution] and incubated for 1–2 h at 37 °C. A TECAN Infinite200 PRO plate reader set at an excitation wavelength of 530 nm and an emission wavelength of 590 nm was used to measure fluorescence on black 96-well plates. The cell viability was calculated as (mean fluorescence of peptide-treated wells/mean fluorescence untreated control wells) × 100%.

### Peptide Stability in CF Patient Sputum

The peptide stability in CF patient sputum was tested as previously described.^52^ Briefly, individual sputum samples from CF patients were pooled and diluted 10-fold with Dulbecco’s phosphate-buffered saline without calcium and magnesium. The suspensions were thoroughly mixed and then centrifuged at 1000 rpm for 10 min. d,l-K_6_L_9_ peptides and LL-37 were added to the supernatant to a final concentration of 100 μM, and the mixture was incubated at 37 °C for several time intervals. The reaction was stopped by adding 10 μL of 10% hydrogen chloride (HCl) to the 55 μL mixture. Residual peptide concentrations were determined via RP-HPLC on a Vydac C18 column (Grace Discovery Sciences, Deerfield, IL) using a linear gradient from 20% to 60% ACN in water (both containing 0.1% TFA) for 50 min. Data are presented as a percentage of peptide compared to the mixture without incubation ± standard errors.

### Killing Assay in CF Sputum

Individual sputum samples from CF patients were pooled and diluted 10-fold with 10 mM phosphate buffer (pH 7.0). The suspensions were thoroughly mixed, centrifuged at 1000 rpm for 10 min, and filtered with a 0.22 μm filter. Supernatants were stored and used for killing assays and fluorescence microscopy, respectively. Then, 10^6^ CFU/mL of *P. aeruginosa* was added to a 10% diluted sputum supernatant, followed by 10 μM peptides. The mixture was incubated at 37 °C for 1 h and then diluted and plated on freshly made LB agar plates.

### Viability of 2 Day Biofilms in the Presence of CF Sputum Visualized by Confocal Microscopy

We visualized the *P. aeruginosa* biofilm using an Olympus FV1000 confocal microscope [60× objective lens (oil)]. A 200 μL volume of 10% diluted sputum supernatant was incubated with a *P. aeruginosa* inoculum (1 × 10^6^ CFU/mL) in eight-well chambered cover glass (Nunc, Thermo Scientific) for 48 h to allow biofilm formation. After treatment with peptides (10 μM) for 1 h, we evaluated the bacterial viability using a Filmtracer live/dead biofilm viability kit (Invitrogen, Life Technologies). Syto-9 (488 nm) marks live bacteria, and propidium iodide (559 nm) marks dead bacteria. For each well, resazurin was added [10 μL of a 0.02% (v/v) solution] and the mixture incubated for 2 h at 37 °C. A plate reader (TECAN Infinite200 PRO) set at an excitation wavelength of 530 nm and an emission wavelength of 590 nm was used to measure the fluorescence on a 96-well plate. The cell viability was calculated as (mean fluorescence of peptide-treated wells/mean fluorescence untreated control wells) × 100%.

### Laboratory Evolution Experiment

The resistance evolution experiment was carried out in 96-well plates at a final volume of 100 μL as previously described^17,19,53^ with minor adjustments. All of the peptides were dissolved in DDW, and 5 μL of the peptide was added to 45 μL of BM2 in a 96-well plate. DDW was used as a negative control. Then, 1 × 10^6^ CFU/mL of three *P. aeruginosa* CF isolates (24, 46, and 82) and PAO1 were suspended in 50 μL aliquots of BM2 and inoculated into each well. The plate was incubated at 37 °C while being agitated for 24 h. Cell growth was monitored after each incubation period by measuring the OD at 600 nm, and 10 μL of the 100 μL culture was transferred to a new 96-well plate containing 90 μL of a fresh BM2 with increasing dosages of antimicrobial peptides. Only populations with the highest concentration were selected for further evolution. If no growth was seen, it was revived from the most recent passage with the appropriate concentration that showed growth. The peptide concentration was increased by 50% in successful growth and was continued for 45 serial transfers. At the end of the experiment, we removed 1.0 μL of bacteria from the well, which led to the highest concentration, that was spread on the LB plate and incubated overnight at 37 °C. Afterward, the MIC assay was performed on a population and a single colony. Next, a single colony was grown in BM2 for three passages without a peptide, and then MIC was determined. Cells during and after the evolution experiments were stored as glycerol stocks at −80 °C.

### Statistical Analysis

Statistical significance was determined using an ANOVA test (*p ≤ 0.05, **p ≤ 0.01, and ***p ≤ 0.001) by Prism and R studio. The results are shown as means ± standard errors of the mean unless indicated otherwise. Experiments were repeated three times (biological repeats) in triplicate or duplicate unless indicated otherwise.

## Supporting Information Available

Susceptibility test parameters of the Phoenix system, mass spectra of peptides, HPLC chromatograms of all peptides, inhibition of CF *P. aeruginosa* biofilm at sub-inhibitory concentrations of AMPs, and degradation of CF *P. aeruginosa* biofilms by AMPs (PDF)

## Abbreviations

ACN: acetonitrile
AMPs: antimicrobial peptides
ASM: artificial sputum medium
BM2: basal medium 2
CF: cystic fibrosis
CFTR: cystic fibrosis transmembrane conductance regulator
CFU: colony-forming units
CV: crystal violet
DCM: dichloromethane
DDW: doubly distilled water
DIC: *N*,*N*′-diisopropylcarbodiimide
DMF: *N*,*N*-dimethylformamide
EPS: extracellular polymeric substances
LB: Luria-Bertani
MALDI-TOF: matrix-assisted laser desorption ionization time-of-flight
MDR: multidrug-resistant
MIC: minimum inhibitory concentration
Oxyma: cyanohydroxyiminoacetate
PBS: phosphate-buffered saline
RP-HPLC: reverse-phase high-performance liquid chromatography
TFA: trifluoroacetic acid
TIS: triisopropylsilane
