# Supplementary material for: Antimicrobial Peptides against Multidrug-Resistant Pseudomonas aeruginosa Biofilm from Cystic Fibrosis Patients
Source: J Med Chem. 2022 Jun 27;65(13):9050–62. doi: 10.1021/acs.jmedchem.2c00270 (PMC9289885; doi:10.1021/acs.jmedchem.2c00270)
Supplement: Supplementary file 1 — jm2c00270_si_001.pdf [file jm2c00270_si_001.pdf]

Supporting Information

Antimicrobial Peptides Against Multidrug-Resistant *Pseudomonas aeruginosa* Biofilm from Cystic Fibrosis Patients

Daniel Ben Hur[†], Gal Kapach[†], Naiem Ahmad Wani[†], Edo Kiper[†], Moshe Ashkenazi[‡,∥], Gill Smollan[§], Natan Keller[⁋, §], Ori Efrati[‡,∥], Yechiel Shai*[,†]

[†]Department of Biomolecular Sciences, Weizmann Institute of Science, Rehovot 76100, Israel

[‡]Pediatric Pulmonary Institute and National CF Center, Edmond and Lily Safra Children's Hospital, Sheba Medical Center, Tel Hashomer, Ramat-Gan 52621, Israel

[∥]Sackler Faculty of Medicine, Tel-Aviv University, Tel-Aviv 69978, Israel

[⁋]The Department of Health Management, Ariel University, Ariel 40700, Israel.

[§] Microbiology Laboratories, Sheba Medical Centre, Tel Hashomer, Ramat-Gan 52621, Israel.

*** Corresponding author**

Email: Yechiel.Shai@weizmann.ac.il

Contents:

Table S1: *P. aeruginosa* CF isolates identification and resistance index
Table S2: Susceptibility test parameters of the phoenix system
Figure S1: Analytical HPLC chromatograms of peptides
Figure S2: Mass spectrum of peptides
Figure S3: Inhibition of clinical CF patient isolates *P. aeruginosa* biofilm formations at sub-inhibitory concentration of AMPs.
Figure S4: D,L-K_6L_9 peptides and LL-37 degrade established clinical isolates CF patient *P. aeruginosa* biofilms

Table S1. *P. aeruginosa* CF isolates identification and resistance index

Isolate No.	Gentamicin	Tobramycin	Amikacin	Ofloxacin	Ciprofloxacin	Ceftazidime	Cefepime	Aztreonam	Piperacillin	Pipera/Tazobactam	Imipenem	Meropenem
24	Resistance	Resistance	Resistance	Resistance	Resistance	Resistance	Resistance	Resistance	Resistance	Resistance	Resistance	Resistance
25	Resistance	Resistance	Sensitive	Resistance	Resistance	Resistance	Resistance	Resistance	Resistance	Resistance	Resistance	Resistance
29	Resistance	Resistance	Resistance	Resistance	Resistance	Resistance	Resistance	Resistance	Intermediate	Intermediate	Resistance	Resistance
40	Resistance	Resistance	Resistance	Resistance	Resistance	Resistance	Resistance	Resistance	Resistance	Resistance	Resistance	Resistance
46	Resistance	Sensitive	Resistance	Resistance	Resistance	Resistance	Resistance	Resistance	Resistance	Resistance	Sensitive	Sensitive
52	Resistance	Resistance	Resistance	Resistance	Resistance	Resistance	Resistance	Resistance	Resistance	Resistance	Sensitive	Sensitive
53	Resistance	Resistance	Resistance	Resistance	Resistance	Resistance	Resistance	Resistance	Resistance	Resistance	Resistance	Resistance
59	Resistance	Sensitive	Sensitive	Resistance	Resistance	Sensitive	Sensitive	Sensitive	Sensitive	Sensitive	Sensitive	Sensitive
71	Resistance	Sensitive	Resistance	Resistance	Resistance	Resistance	Resistance	Resistance	Resistance	Resistance	Resistance	Resistance
72	Resistance	Resistance	Resistance	Resistance	Resistance	Resistance	Resistance	Resistance	Resistance	Resistance	Resistance	Resistance
82	Resistance	Resistance	Sensitive	Sensitive	Sensitive	Resistance	Resistance	Resistance	Resistance	Resistance	Resistance	Resistance
94	Resistance	Resistance	Resistance	Resistance	Resistance	Resistance	Resistance	Resistance	Resistance	Resistance	Resistance	Resistance
95	Resistance	Resistance	Resistance	Resistance	Resistance	Resistance	Resistance	Resistance	Resistance	Resistance	Resistance	Resistance
99	Sensitive	Sensitive	Sensitive	Sensitive	Sensitive	Sensitive	Sensitive	Sensitive	Sensitive	Sensitive	Sensitive	Sensitive
172	Resistance	Resistance	Resistance	Resistance	Resistance	Resistance	Resistance	Resistance	Resistance	Resistance	Resistance	Resistance
238	Resistance	Resistance	Resistance	Resistance	Resistance	Resistance	Resistance	Resistance	Resistance	Resistance	Resistance	Resistance
251	Resistance	Resistance	Resistance	Resistance	Resistance	Resistance	Resistance	Resistance	Resistance	Resistance	Resistance	Resistance
629	Sensitive	Resistance	Sensitive	Sensitive	Resistance	Resistance	Resistance	Resistance	Resistance	Sensitive	Resistance	Resistance
895	Resistance	Resistance	Resistance	Resistance	Resistance	Resistance	Resistance	Resistance	Resistance	Resistance	Resistance	Resistance
896	Resistance	Sensitive	Sensitive	Sensitive	Sensitive	Sensitive	Sensitive	Sensitive	Sensitive	Sensitive	Sensitive	Sensitive
995	Resistance	Resistance	Resistance	Resistance	Resistance	Resistance	Resistance	Sensitive	Sensitive	Resistance	Resistance	Resistance

■ - Sensitive ■ - Intermediate ■ - Resistance

Identification and susceptibility were conduct by BD Phoenix™ automated identification and susceptibility testing system

Table S2: Susceptibility test parameters of the Phoenix system

Drug class	Drug name	Drug range	MIC (S)	MIC (I)	MIC (R)
Aminoglycoside	Gentamicin	0.25-16	≤4	8	≥16
Aminoglycoside	Tobramycin	0.12-16	≤4	8	≥16
Aminoglycoside	Amikacin	0.5-64	≤16	32	≥64
5-Fluoroquinolone	Ofloxacin	0.25-8	≤2	4	≥8
5-Fluoroquinolone	Ciprofloxacin	0.25-4	≤1	2	≥4
Cephem	Ceftazidime	0.5-64	≤8	16	≥32
Cephem	Cefepime	0.5-64	≤8	16	≥32
Monobactam	Aztreonam	0.5-64	≤8	16	≥32
B-Lactam Pen	Piperacillin	0.5-128	≤16	32-64	≥128
B-Lac/B-Lac. Inh	Pipera/ Tazobactam	0.5/4-128/4	≤13	32-64	≥128
Carbapenem	Imipenem	1-16	≤14	4	≥8
Carbapenem	Meropenem	0.25-16	≤15	4	≥8

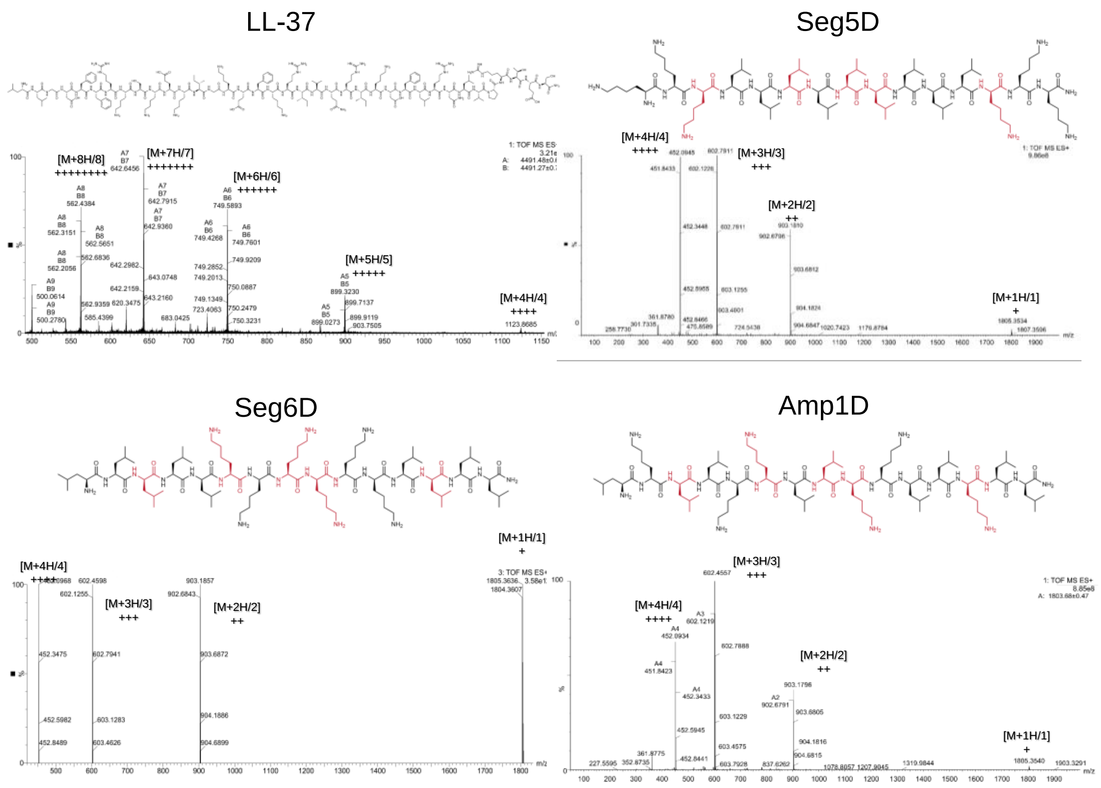

Figure S1: Mass spectrum of peptides

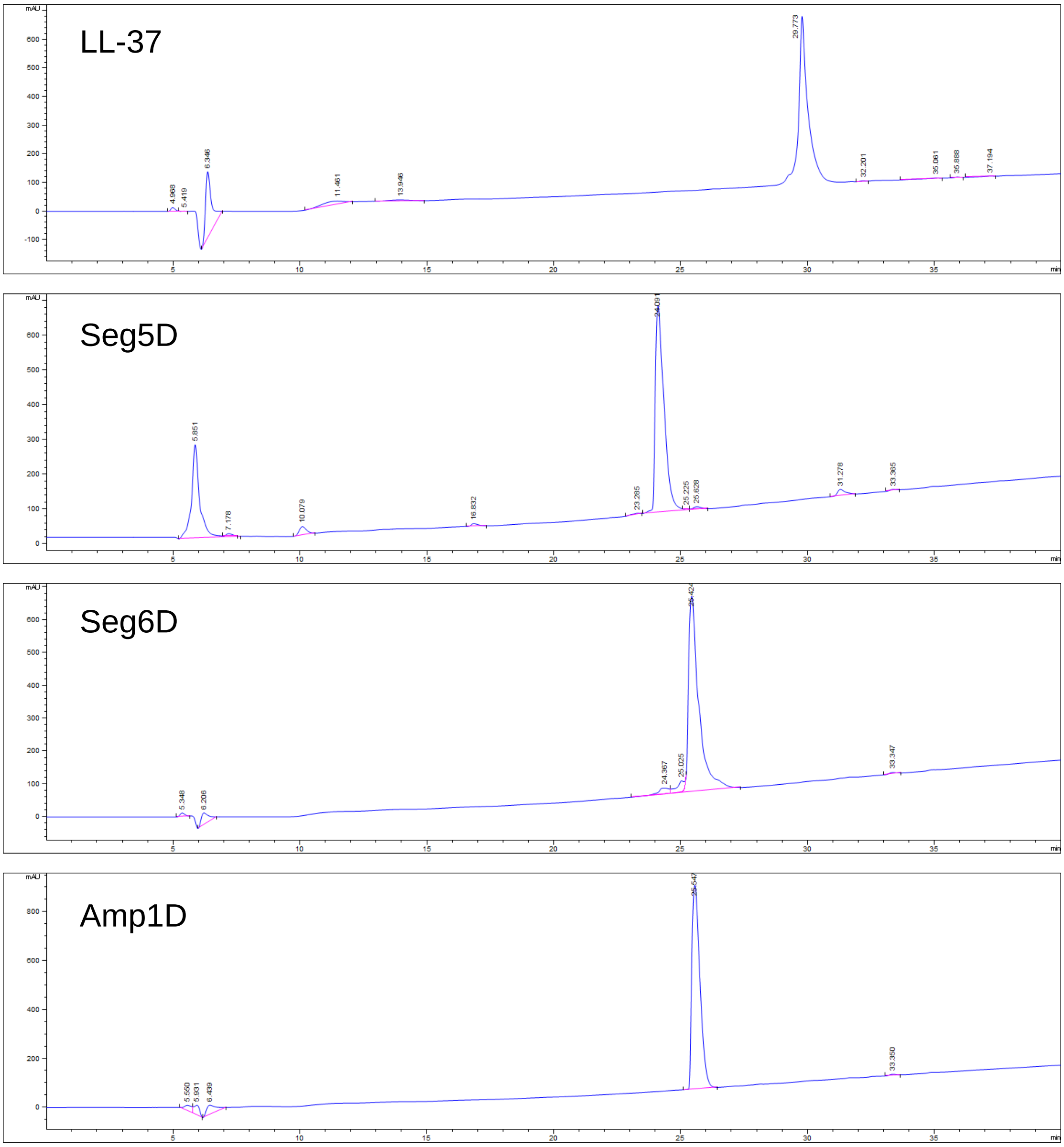

Figure S2: Analytical HPLC chromatograms of peptides

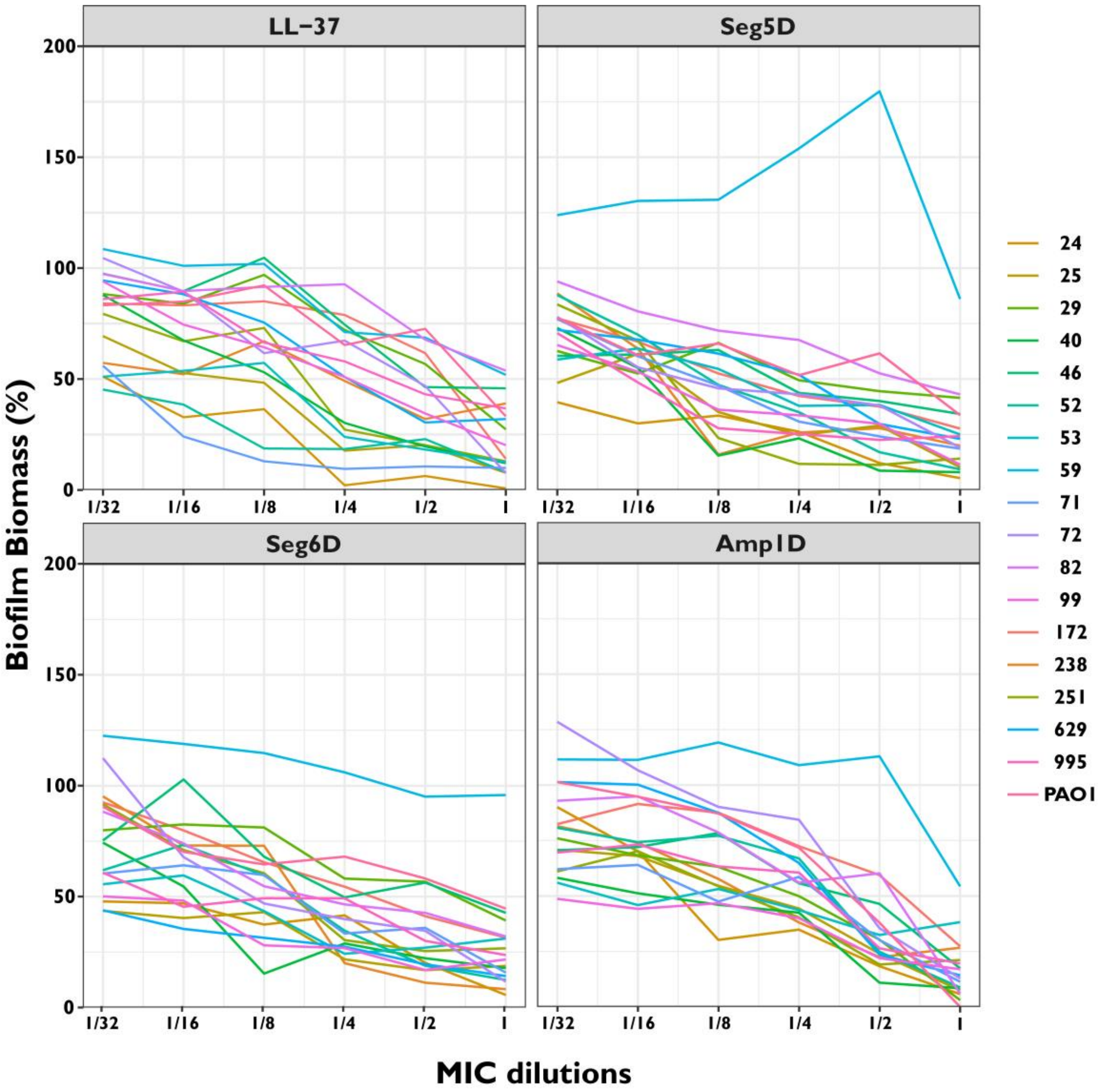

Figure S3. Inhibition of clinical CF patient isolates *P. aeruginosa* biofilm formations at sub-inhibitory concentration of AMPs. *P. aeruginosa* bacteria were incubated for 24 h in the presence of AMPs (at MIC dilutions). Surface-associated biofilm after treatment was examined using 0.1% CV staining followed by absorbance measurements at 590 nm. Results are reported relative to untreated biofilm. Each graph represents an isolated sample (shows in the left bottom of each graph). Background measurement with no added bacteria were preformed as blank.

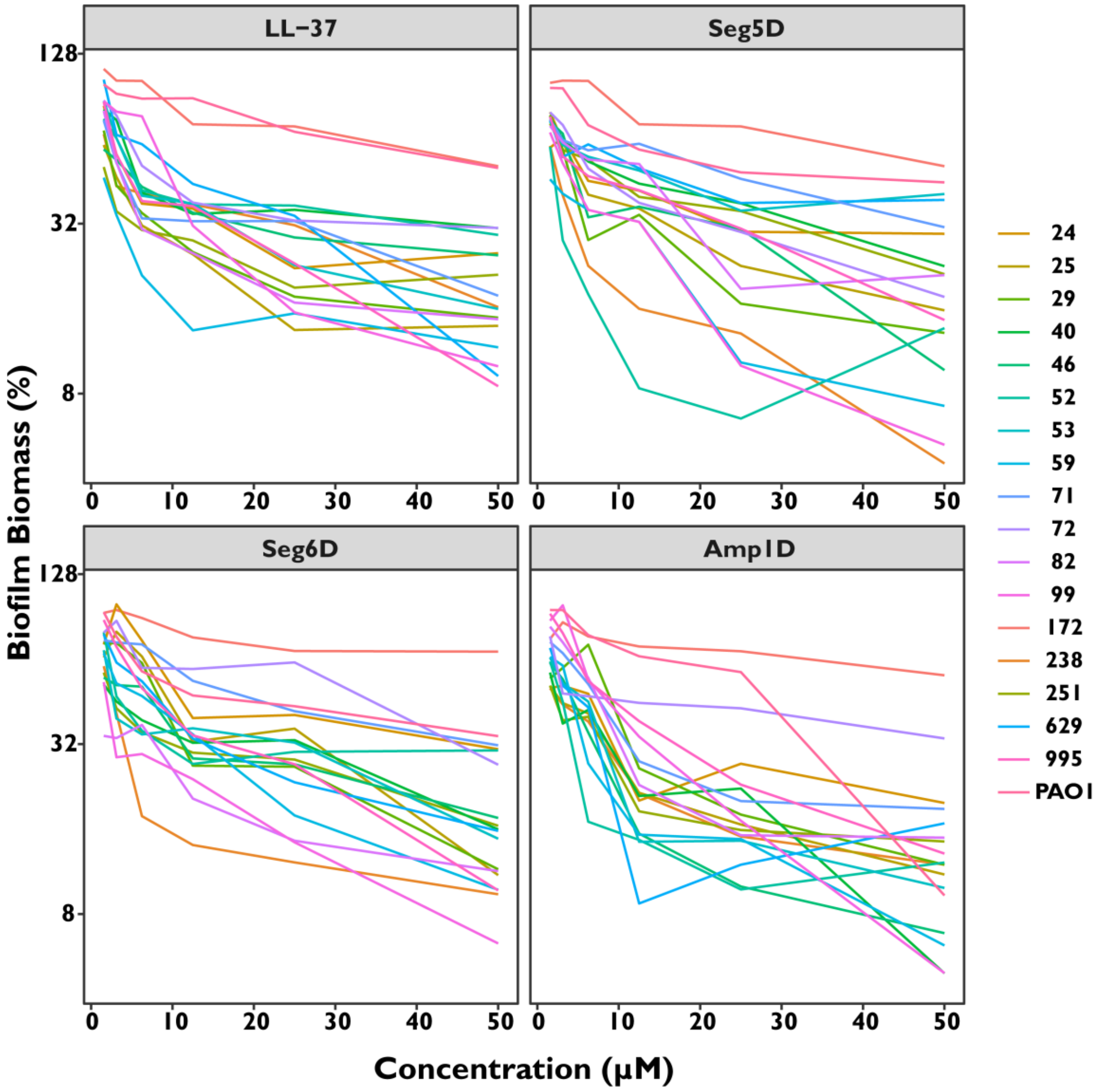

Figure S4. D,L-K_6L_9 peptides and LL-37 degrade established clinical isolates CF patient *P. aeruginosa* biofilms. *P. aeruginosa bacteria* were allowed to grew for 24-h and treated for 1-hour with peptides in a serial dilution concentrations. Surface-associated biofilm after treatment, examined using 0.1% CV staining followed by absorbance measurements at 590 nm. Results are reported relative to untreated biofilm. Each graph represent isolated sample (shows in the left bottom of each graph). Background measurement with no added bacteria were preformed as blank
